# Do cash or digital payment modalities affect community health worker performance? – a case study of a remote refugee settlement in Western Uganda

**DOI:** 10.1080/16549716.2024.2375867

**Published:** 2024-08-23

**Authors:** Michael T Wagaba, David Musoke, Charles Opio, Arthur Bagonza, Juliet Aweko, Hajarah Nakitende, Alex Mulyowa, Michael Ediau, Peter Waiswa, Elizabeth Ekirapa-Kiracho

**Affiliations:** a Department of Community Health and Behavioral Sciences, Makerere University School of Public Health, Kampala, Uganda; b Department of Disease Control and Environmental Health, School of Public Health, Makerere University, Kampala, Uganda; c Department of Health Policy, Planning and Management, School of Public Health, Makerere University, Kampala, Uganda

**Keywords:** Healthcare delivery, performance-motivation, digital and cash payments, vulnerable communities, community health workers

## Abstract

**Background:**

There is inadequate evidence about the influence of digital and cash payment modalities on the performance of Community Health Workers (CHWs) in underserved communities, such as refugee settlements.

**Objective:**

To compare the performance of CHWs when paid in cash or digitally in Kyaka II refugee settlement, Uganda.

**Methods:**

A comparative cross-sectional mixed methods design was used. Secondary data comprising 247 CHW reports during a six-month period of cash and digital payments were analyzed using Stata v14. Eleven focus group discussions, four in-depth interviews, and ten key informant interviews were conducted among the settlement stakeholders to explore perceptions of the payment methods. Qualitative data were analyzed thematically using Atlas.ti v9.

**Results:**

CHWs performed better when paid cash than digital payments (*t* = 5.28; df = 246; *p* < 0.001). During the cash payment period, at least secondary education (APR 1.71 CI: 1.14–2.58) and having a side occupation (APR 1.58; CI: 1.13–2.21) were positively associated with performance. For digital payments, being male (APR 0.58; CI: 0.34–0.98), serving longer than 9 years (APR 0.87; CI: 0.82–0.93), and being allocated more than 60 households per month (APR 0.31; CI: 0.19–0.52) were negatively associated with CHW performance. Qualitative data revealed that most stakeholders preferred cash due to inconsistent and delayed digital payments.

**Conclusion:**

CHWs preferred and performed better with cash payments because digital payments were associated with delays and payment shortfalls that demotivated them. Implementers should invest towards averting digital payment shortfalls in remote settings to enhance CHW motivation and performance.

## PAPER CONTEXT

- **Main findings:** Regarding the influence of payment modalities on Community Health Workers’ performance, this study found that community health workers were motivated to perform better when paid with cash compared to digital payments.
- **Added knowledge:** Cash payments offer better reliability than digital payments in refugee settlements, which underscores the need to understand further the barriers to digital payments, particularly in hard-to-reach areas.
- **Global health impact for policy and action:** Health systems adapting to digital payments should consider a gradual transition through a hybrid approach that includes cash and digital payments while addressing the challenges associated with digital payments, especially in refugee settings and similar resource-constrained contexts.

## Background

Health workforce motivation is critical to enhancing the performance and productivity of Community Health Workers (CHWs), given their essential role in healthcare delivery, especially in remote and low-resource settings [1,2]. CHWs are paraprofessionals with basic healthcare training selected by the community to primarily provide essential health services. They are usually well versed with the local culture and are fluent in the local language [3,4]. In Uganda, CHWs work as members of health teams at the village level, locally known as Village Health Teams (VHTs). They bridge the gap between under-served households and the formal health system, especially in low- and middle-income countries (LMICs) [5]. CHWs also play a crucial role in improving the health of vulnerable populations such as refugees [6,7] who face diverse socioeconomic challenges [8,9]. Unfortunately, many health systems are yet to consider formalizing CHWs’ monetary incentives/remunerations [10–12]. In settings where CHWs receive monetary incentives, inequalities in payments have often been reported, which may hinder optimal performance, subsequently leading to poor health service delivery [13,14].

Furthermore, CHWs encounter various challenges while executing their duties, including a heavy workload, low education levels, inadequate incentives, poor supervision, and facilitation [15–22]. The most common challenge, however, is poor monetary incentives, including insufficient and delayed payments, which leads to the CHWs diverting their time to other duties to find income to sustain their families [23–26]. Monetary incentives for CHWs play a significant role in the success of vaccination campaigns and contribute to positive outcomes in some settings. Financial incentives boost immunization rates by covering transportation and coordination costs, especially in resource-limited areas [27]. Other factors that influence performance include the CHW’s gender, experience, and workload [28–33]. Digital payments have been fronted to reduce payment and expenditure irregularities [34–36]. However, its application among frontline health workers is still low, with limited evidence on its outcomes and associated determinants [37].

Uganda is the third largest refugee-hosting country worldwide, with the largest refugee population in Africa of almost two million refugees spread over 13 settlements and urban settings [38]. As a result, there is increased pressure on the available resources, especially the health workforce, due to the accumulating number of refugees from the neighboring war-ravaged Democratic Republic of Congo (DRC) [39]. However, these gaps are buffered through CHW activities, including community mobilization and health surveillance through household visits, immunization, referrals, maternal and child health promotion, and basic disease management. Generally, the CHW strategy has contributed to the improved health status of Ugandans despite the implementation challenges that have been encountered [40].

In Uganda, CHWs’ performance is assessed based on their monthly reports, premised on fulfilling their assigned tasks, including household visits, data reporting quality, and proportion of referrals [4]. Evidence indicates that less than 40% of the CHWs in the Uganda refugee host communities meet the performance standards [41–43]. Poor health workforce performance in resource-constrained settings has been linked with absenteeism, strikes, and dropouts [44–47], resulting from poor financial incentives [48]. However, similar evidence in the context of refugee settlement remains limited. Furthermore, many studies and campaigns have focused on the adequacy of the amount of compensation and the varying effects [48–50]. Information on the modes of payment is not well documented, yet it could elucidate challenges such as delayed and missing payments that are critical to CHW motivation. Moreover, since CHWs are conventionally unsalaried, there is limited evidence on their mode of monetary compensation. This dearth of knowledge on how the choice of payment modes influences CHWs’ performance is more pronounced in refugee settings, with an often neglected vulnerable population usually regarded as secondary citizens [8].

To address the performance challenges, the humanitarian agencies in the Kyaka II refugee settlement, such as African Humanitarian Action (AHA) and Medical Teams International (MTI), operate an incentive-based system. This mode of operation entails paying CHWs a monthly stipend upon accomplishing their assigned targets. However, these health workers are affected by delayed, missing and incomplete payments partly due to bureaucratic processes. Such challenges demotivate them, leading to absenteeism, strikes, drop-outs, and poor performance [36,48,51]. Consequently, the implementing partners introduced digital payment methods; however, their outcome on the performance of CHWs has not been determined. Therefore, in this study, we assessed how digital and cash payment modalities influenced the CHWs’ performance in a multi-national refugee settlement where the CHWs were paid using both cash and digital means.

## Methods

### Study setting

The study was conducted in Kyaka II Refugee Settlement located in Kyegegwa district in Southwestern Uganda. The settlement consists of nine zones and 26 villages, with Ugandan nationals living within and in areas neighboring the settlement. The settlement hosts an estimated population of 121,934 refugees and asylum seekers in 32,883 households. Most refugees in Uganda are from the DRC (115, 346), Burundi (3,233) and Rwanda (3,104) [52]. They receive professional healthcare from only three Health Center III facilities and six medical outposts [53]. This settlement was selected because it operates cash and digital payment methods for CHW remuneration. The CHWs are paid in cash for the first 6 months of the year and then digitally through mobile money in the subsequent 6 months.

### Study participants

The study population comprised CHWs in the Kyaka II Refugee settlement who were paid to conduct community health promotion and surveillance activities using cash and digital payment methods. CHWs from the host communities were excluded since implementing partners did not provide monetary incentives. Study participants and data sources included the CHWs and their service records over a period of 12 months in 2021. Key informants included community leaders, officials from health facilities, representatives of implementing partner organisations, and the United Nations High Commission for Refugees (UNHCR).

### Study design

This study had a comparative cross-sectional design that employed sequential explanatory mixed methods. The quantitative methods were used to determine the CHW performance levels and factors that affect performance. In contrast, the qualitative methods were used to explore the stakeholder perspectives on CHW performance and its determinants.

### Sample size determination

The Kyaka II Refugee settlement had a total of 308 CHWs. The desired sample size generated using the finite population formula was 160. To increase the power of the study, we included records for all the eligible participants for the quantitative data (247). The excluded 61 records belonged to non-refugee CHWs from the host communities. This sample size was more significant than the 160 generated using the finite population formula, so we are confident that the study had sufficient power. For the qualitative phase of the study, we purposively sampled participants for FGDs, IDIs, and KIIs to diversify the information sources [54,55], while allocating adequate time to the participants [56] to enable information saturation. For the IDIs and KIIs, we selected participants with knowledge and experience regarding CHW operations. They included two (2) CHW leaders for in-depth interviews (IDIs) and ten (10) key informants who comprised local leaders (2), IPs (4), health facility staff (3), and the UNHCR representative (1). We also conducted seven (7) FGDs, each group comprising eight participants purposively selected and categorized based on variables of interest, including sex (female CHWs) (1); reporting method (CHWs using paper) (1) or digital (1); zone performance: CHWs from the best performing zone (1); CHWs from the poorest performing zone (1); and mixed groups (CHWs from all categories) (2) (Table 1).

**Table 1.** Selection of qualitative study participants.

Method (Quantity)	Category (quantity)	Stratification
CHW FGD (7)		
	Sex (1)	Female
	Reporting method (2)	Paper and mHealth users
	Refugee Zone performance* (2)	Worst and best performers
	Mixed groups (2)	All categories represented
Key Informant Interviews (10)		
	Local leaders (2)	National and refugee community leader
	Implementing partners (4)	Health implementing partner representatives
	Health facility (3)	Health Centre III and II representatives
	OPM representative (1)	Commandant’s office
In-Depth Interviews (2)	CHW leaders (2)	Two refugee CHW leaders
Total	7 FGDs, 10 KIIs; 2 IDIs	

*Zone performance was based on the implementing partner records of the CHW performance.

### Data collection tools and procedures

#### Quantitative data

CHW monthly reports submitted by each of the 247 CHWs in 2021 were obtained from the databases of the implementing partners (IPs). Four research assistants from the School of Public Health, Makerere University, who are experienced in refugee settlement data collection and ethical conduct, assisted with data collection. They used Epicollect and the KoboCollect data collection software, guided by a CHW performance indicator data extraction sheet. Secondary data on the CHWs’ performance indicators and characteristics were collected from monthly reports stored in the KoboCollect and Epicollect databases. The data were accessed with permission from the Office of the Prime Minister (OPM) and the Implementing Partners. The research assistants extracted the secondary data from the IP databases and organized it into a single Microsoft Excel dataset with all the relevant study variables and metadata.

#### Qualitative data

IDI, KII and FGD guides were used to collect qualitative data. The tools were unstructured, comprising open-ended questions with probes informed by previous literature [15–36,57] and prior orientation on the settlement’s context to clarify responses. Voice recorders were used to capture the audio data. Telephone calls were made to the individual participants to schedule interviews and discussions. All participants verbally consented to the audio recording of the sessions. All qualitative interviews and discussions lasted 45 min on average. The interviews and discussions were conducted in English or Swahili by the principal investigator, study coordinator, and two research assistants. The FGDs and IDIs were conducted in safe, private locations within the Kyaka II refugee settlement, while KIIs were conducted at the respective implementing partners’ offices.

### Quality assurance

All data collection tools were pre-tested before data collection, with a seven-member FGD and two key informant representatives in Kaborogota – a zone that did not contribute to the qualitative study participants. A three-day training comprising sessions on ethical conduct, translation, basics of qualitative data collection and analyses and key concepts in the guides was held for the research assistants. All qualitative data collection sites ensured the privacy and safety of the participants. Non-consenting participants were replaced through the same sampling methods. The principal investigator supervised the data collection and held debriefing sessions with the study coordinator before, during, and after the data collection and analysis. The quantitative data entries were checked and corrected daily for accuracy and completeness.

### Data management and analysis

#### Quantitative

Data from the electronic archives and the CHW registers were extracted using two Microsoft Excel sheets to disaggregate the cash and mobile money payment periods, then imported into Stata version 14 software (Statacorp, 2015, College Station; USA) for analysis. In univariate analysis, we obtained means and standard deviations for continuous variables and frequencies and proportions for discrete and categorical variables. Overall performance was either optimal or sub-optimal; optimal performance was equivalent to scoring over 80% in at least four of the five selected performance indicators. A CHW who obtained an average score (over the 6-month periods disaggregated by the payment method) of at least 80% in each of the chosen indicators was categorized as having obtained optimal performance for the indicator assessed. Consequently, the performance between the two groups was compared.

The selected indicators included the proportion of households visited and reported monthly (the denominator was the monthly target allotted to a CHW), the proportion of under-five-year-olds within the CHW’s catchment area screened for malnutrition, supervision and training meetings attended, reporting quality as objectively scored by two Public Health Assistants, and proportion of cases referred to the health facilities per month. Previous CHWs’ performance measurement studies informed the selected performance indicators and cut-offs [4,20,41], triangulated by information from interviews with the settlement’s stakeholders (representatives of implementing partners, UNHCR, health facilities, and CHWs) to guide context relevance and specificity.

At the bivariate level, all variables were independently analyzed using Chi-square or Fisher’s exact tests for associations between categorical variables and CHW performance. We obtained prevalence ratios to establish the strength of the associations. The prevalence ratio was selected instead of odds ratios because the study outcome, optimal CHW performance under digital and cash payment modes, was approximately 20% and 40%, respectively. Using odds ratios with a study outcome greater than 10% would have exaggerated the strength of the association, and confounders would not have been appropriately controlled [18,58–60]. Associations with a p-value less than 0.25 at the bivariate level were considered to qualify for multivariable analysis. At the multivariable level, the modified (robust variance) Poisson regression analysis was used to control for confounding to confirm statistical significance [61], at a *p-*value of less than 0.05 and the corresponding 95% confidence interval (CI).

#### Qualitative

For the qualitative study, the audio data were collected in English and Swahili, safely stored with computer encryptions and subsequently transcribed verbatim. The files in Swahili were translated to English to foster uniformity during analysis. The transcripts were proofread by two independent researchers and two analysis team members to check for the coherence of the information and its alignment with the study subject. The team conducted a thematic analysis identifying patterns from the information provided in the transcripts. A comprehensive codebook was created outlining thematic areas from the qualitative data. Themes were developed inductively using open and axial coding. The codebook and transcripts were subsequently uploaded to Atlas.ti software (v9) for coding and analysis. The results were subsequently aligned to complement the quantitative findings.

### Ethical considerations

Permission was obtained from the Prime Minister’s Office to conduct research within the refugee settlement. Additionally, approval was obtained from the Higher Degrees Research and Ethics Committee of Makerere University School of Public Health (Protocol number: 059). Written informed consent was obtained from participants before they participated in the study. Participants were assured of autonomy, confidentiality, and anonymity and were interviewed in a secure and private environment to avoid injury or harm. Their permission was sought and received to audio record the sessions.

## Results

Participants’ characteristics Overall, most of the CHWs 71.3% (176/247) were male, 61.7% (153/247) were less than 35 years old, 76.1% (188/247) had served between one and four years, and 72.3% (179/247) had attained a minimum of a secondary education level. The majority, 90.3% (223/247), had been allocated more than 60 households to visit each month, and 83.0% (205/247) were married, as shown in Table 2.

**Table 2.** Community health workers’ sociodemographic and work characteristics (*n* = 247).

	Cash Payment Period		Digital Payment Period			
	n	%	N	%	χ2	*p* value
**Variable**					0.65	0.42
**Sex**						
Male	176	71.3	177	71.7		
Female	71	28.7	70	28.3		
**Age categories (years)**					0.097	0.76
≤35	153	61.7	152	61.5		
>35	94	38.3	95	38.5		
**Mean Age (**±SD)	33.7 (±9.3)		33.9 (±9.5)			
**Education level**					0.91	0.34
Primary and below	68	27.7	69	27.9		
Secondary and above	179	72.3	178	72.1		
**VHT experience (years)**					2.12	0.72
1–4	188	76.1	187	75.7		
5–9	51	20.7	52	21.1		
10+	8	3.2	8	3.2		
**Phone ownership**					0.69	0.41
Yes	134	54.5	135	54.7		
No	113	45.5	112	45.3		
**Households allocated**					1.46	0.22
≤60	24	9.7	24	9.7		
Above 60	223	90.3	223	90.3		
**Marital status**					0.15	0.70
Married	205	83.0	205	83.0		
Single	42	17.0	42	17.0		
**Side jobs**					0.83	0.36
None	103	41.5	103	41.7		
With side jobs	144	58.5	144	58.3		
**Trainings**					3.18	0.08
0–4	136	55.1	191	77.3		
5–9	111	44.9	56	22.7		
**Supervision Frequency**					1.41	0.24
Weekly	183	74.1	183	74.1		
Monthly	64	25.9	64	25.9		

### Community health worker performance

**Figure 1.**
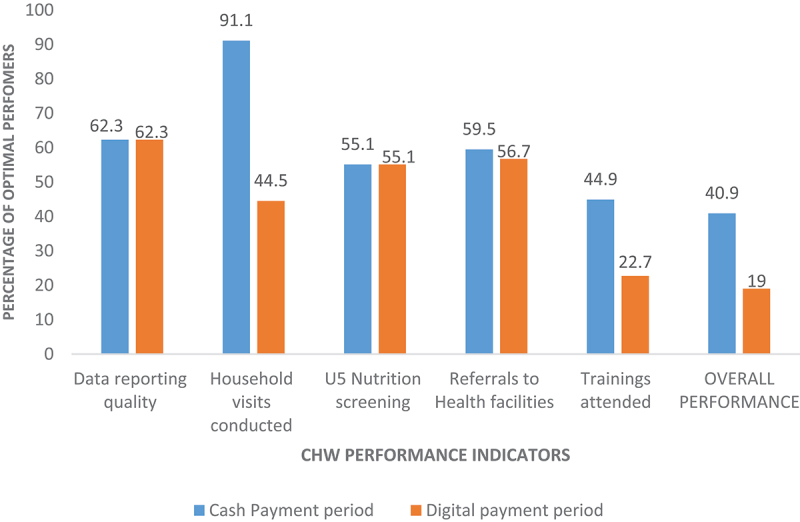
Community health worker performance indicator scores.

Overall, CHW performance was better during the cash payment period than during the digital payment period. We found that the number of CHWs with optimal performance when they were paid in cash, 40.9% (101/247), was twice the number of that when they were paid with mobile money, 19.0% (47/247) (Figure 1). A paired t-test confirmed that the difference in performance between the two periods was statistically significant (*t* = 5.28; df = 246; *p* < 0.001).

Figure 1 shows that during the period of cash payments, the proportion of CHWs who attained the optimal number of household visits (91.1%, 225/247), and trainings attended (44.9%, 111/247) were double that during the period of mobile money payments (44.5%, 110/247 and 22.7%, 56/247, respectively). Finally, more CHWs, 59.5% (147/247), achieved the set referrals to health facilities target of at least 80% when they received cash payments compared to when they were paid using mobile money, 56.7% (140/247). There was no substantial variability between other performance indicators across the two payment periods. The qualitative study participants attributed the better performance during the cash payment period to the enhanced motivation of the CHWs. Most FGD participants reported CHWs’ increased morale to perform when paid using cash because it was prompt and assured. The CHWs received their money after each activity without marked payment delays or irregularities.

We would say that at least we prefer cash to this mobile money because, with cash, we sign and get the money there and then [uhm], but this one [mobile money], we signed, but up to now, nothing. We don’t know anything about when it will come. *–* FGD_CHW_ Worst performing zone

### Factors associated with Community Health Worker performance across the payment modalities

When the CHWs received cash payments, those with a secondary level of education and above performed almost twice as well as those with a primary education level or lower (APR = 1.71; 95%CI:1.14–2.58). On the other hand, the level of education did not significantly influence the CHW performance when being paid digitally (Table 3). This finding was affirmed by the responses from the qualitative study positing that CHWs with a higher education level quickly attained the skill to deliver health services and were more closely bonded with the implementing partners. However, they were more dedicated and motivated to perform better when they received their financial incentives promptly, and this was through cash payments. Missed and delayed mobile money payments had a negative impact on the value of money and the morale of community health workers, particularly those who were more educated. As a result, they prioritized other jobs, giving less attention to community health work. As two FGD participants said:

**Table 3.** Factors associated with community health worker performance during the cash payment period (*n* = 247).

	VHT performance					
Variable	Optimal N = 101 f (%)	Suboptimal N = 146 f (%)	CPR (95% CI)	p-value	APR (95% CI)	*p* value
**Sex**						
Female	32 (31.7)	40 (27.4)	1		1	
Male	69 (68.3)	106 (72.6)	0.89 (0.64–1.22)	0.82	0.76 (0.56–1.05)	0.10
**Age categories**						
<35	59 (58.4)	94 (64.4)	1		1	
>35	42 (41.6)	52 (35.6)	1.16 (0.86–1.57)	0.34	1.27 (0.94–1.71)	0.12
**Education level**						
Primary and below	20 (19.8)	48 (32.9)	1		1	
Secondary and above	81 (80.2)	98 (67.1)	1.54 (1.03–2.30)	0.04*	1.71 (1.14–2.58)	0.01*
**VHT experience (years)**						
1–4	79 (78.2)	110 (75.3)	1		1	
5–8	20 (19.8)	30 (20.6)	0.96 (0.65–1.40)	0.85	0.97 (0.66–1.42)	0.88
10+	2 (2.0)	6 (4.1)	0.60 (0.18–2.01)	0.53	0.62 (0.20–1.87)	0.40
**Phone Ownership**						
No	45 (44.5)	68 (46.6)	1		1	
Yes	56 (55.5)	78 (53.4)	1.04 (0.78–1.42)	0.76	0.96 (0.71–1.29)	0.77
**Households allocated**						
<60	14 (13.9)	10 (6.8)	1		1	
Above 60	87 (86.1)	136 (93.2)	0.67 (0.45–0.97)	0.04*	0.75 (0.51–1.09)	0.13
**Marital status**						
Single	15 (14.9)	27 (18.5)	1		1	
Married	86 (85.1)	119 (81.5)	1.17 (0.76–1.82)	0.47	1.26 (0.81–1.96)	0.30
**Other occupation**						
None	31 (30.7)	71 (48.6)	1		1	
With side occupations	70 (69.3)	75 (51.4)	1.59 (1.13–2.23)	0.01*	1.58 (1.13–2.21)	0.01*
**Supervision Frequency**						
Monthly	27 (26.7)	37 (25.3)	1		1	
Weekly	74 (73.3)	109 (74.7)	0.96 (0.68–1.34)	0.81	0.90 (0.64–1.27)	0.56

*Statistically significant values at *p* ≤ 0.05; 95% Confidence Interval.

I’m educated, and that’s why when I receive it [payment] in hand, I start using it to generate income. But if it doesn’t come in cash, and it delays [like mobile money], then I cannot use it for generating anything FGD_CHW_Mixed

I studied to get benefits in life and to get skills in order to prosper in life. When you get that direct cash, you feel happy about having attended school. You go to work when you know what you’re doing; that’s why motivation increases more when you get it by cash *–* FGD_CHW_Mixed

Similarly, CHWs with side jobs performed 1.5 times better than those without side jobs (APR = 1.58; 95%CI: 1.13–2.21) during the cash payment period, but there was no significant association (APR = 1.40; 95%CI: 0.81–2.42) when CHWs were paid digitally. Discussions with CHWs alluded to similar findings, stating that since cash payments were prompt and reliable, CHWs with side jobs were not diverted from fulfilling their obligations to the community since they received prompt payment for their community service. On the other hand, digital payments were often delayed and, in some cases, not fulfilled; hence, they tended to neglect their work and paid more attention to their side jobs, which paid them in cash.

Everyone concentrates where he/she gets benefits; you may have two jobs. You realize this one is more important than the other because you get benefits at the right time. I don’t think you can go for that job where you don’t get benefits at the right time, leaving that one where you get them on time. We are all human beings you can try it and see it; you concentrate on that one that is giving you money on time. *–*FGD CHW_ Best performing zone

In another FGD, a participant had this to say:

The reason why they are happy about doing other things is that whatever else they do, they receive cash, and it is prompt and helps their children. Whereas, when the VHT payment is through mobile money and it is delayed for like two months, we will be paid for one month. *–* FGD CHW_ Best performing zone

Conversely, during the period of digital payment, the performance level of the male CHWs was found to be half that of the female CHWs (APR = 0.58; 95%CI: 0.34–0.98); however, there was no significant association between performance and the CHWs’ gender when they were paid in cash. The qualitative findings rationalized the quantitative results when participants mentioned that women were more resilient and tenacious amidst various challenges of digital payment irregularities generally associated with reduced morale/motivation. While male CHWs quickly lost morale to work due to the poor and delayed monetary incentives, women persisted and worked with dedication. Hence, they performed better than their male counterparts during this period.

Women listen more than men, and we are patient. A lot of problems arise and we suffer, but we try to maintain our patience and wait for the mobile money even though we are not happy with it. – FGD CHW_ Best performing zone

Another FGD reinforced this by observing that

The truth is people are different; women are different from men because men have a lot of plans and work for their families. It means he can’t concentrate on that job where he will get the soap that doesn’t come on time. He’d rather go to the host communities to work for something to eat with the family. *–* FGD CHW_ Best performing zone

While receiving digital payments, CHWs who had served for more than 10 years performed poorer than their counterparts with less than 10 years of experience (APR = 0.88; 95%CI: 0.82–0.93). There was no significant association between the CHWs’ experience and their performance while they received cash payments. In-depth interviews with CHWs intimated that the longer CHWs served, the less enthusiastic and dedicated they became towards their CHW duties. Nevertheless, they continued to serve due to the incentives tagged to the job. However, their motivation to serve further deteriorated when the incentives were not delivered as promised. As one CHW leader mentioned in an interview:

These people [long-serving CHWs] are just used to the work, and those are the people who used to be serious leaders; sometimes, they don’t work when there is no money. Because payment from mobile money is regarded as money that we are not receiving because it is not visible [missed payments], and because it is delayed. IDI_CHW_Leader

Generally, CHWs with more than 60 households performed poorer than those with fewer households, but the difference in performance was more significant when they were paid digitally. The CHWs serving more than 60 households were less likely to perform optimally than their counterparts serving 60 or fewer households (APR = 0.34; 95%CI: 0.19–0.61). The qualitative findings complemented these results by indicating that besides the huge CHWs’ workload of more than 60 households, the payment irregularities linked to mobile money were particularly deleterious to the CHWs’ zeal to attain the household visit targets (Table 4). One KII participant mentioned:

**Table 4.** Factors associated with community health worker performance during the digital payment period (*n* = 247).

	VHT performance					
Variable	Optimal N = 47 n (%)	Suboptimal N = 200 n (%)	CPR (95% CI)	*p-*value	APR (95% CI)	*p-*value
**Sex**						
Female	19 (40.4)	51 (25.5)	1		1	
Male	28 (59.6)	149 (74.5)	0.58 (0.34–0.97)	0.04*	0.58 (0.34–0.98)	0.04*
**Age categories**						
<35	30 (63.8)	122 (61.0)	1		1	
>35	17 (36.2)	78 (39.0)	0.91 (0.53–1.55)	0.72	1.00 (0.58–1.71)	0.99
**Education level**						
Primary and below	9 (19.2)	60 (30.0)	1		1	
Secondary and above	38 (80.8)	140 (70.0)	1.64 (0.83–3.21)	0.51	1.57 (0.84–2.94)	0.16
**CHW experience (years)**						
1–4	37 (78.7)	150 (75.0)	1			
5–8	10 (21.3)	42 (21.0)	0.97 (0.52–1.82)	0.93	1.15 (0.61–2.14)	0.67
9+	0 (0.0)	8 (4.0)	0.87 (0.83–0.91)	<0.001*	0.88 (0.82–0.93)	<0.001*
**Phone ownership**						
No	17 (36.2)	95 (47.5)	1		1	
Yes	30 (63.8)	105 (52.5)	1.46 (0.85–2.51)	0.17	1.47 (0.87–2.47)	0.15
**Households allocated**						
<60	12 (25.5)	12 (6.0)	1		1	
Above 60	35 (74.5)	188 (94.0)	0.31 (0.19–0.52)	<0.001*	0.34 (0.19–0.61)	<0.001*
**Marital status**						
Single	10 (21.3)	32 (16.0)	1		1	
Married	37 (78.7)	168 (84.0)	0.76 (0.41–1.40)	0.38	0.90 (0.46–1.75)	0.75
**Other occupation**						
None	15 (31.9)	88 (44.0)	1		1	
With side occupations	32 (68.1)	112 (56.0)	1.52 (0.87–2.67)	0.14	1.40 (0.81–2.42)	0.22
**Supervision Frequency**						
Monthly	11 (23.4)	53 (26.5)	1		1	
Weekly	36 (76.6)	147 (73.5)	1.14 (0.62–2.11)	0.67	1.01 (0.53–1.90)	0.98

*Statistically significant values at *p* ≤ 0.05; 95% Confidence Interval.

Now, by the Ministry of Health standard, it was supposed to be one VHT for 25, but currently, one VHT has around 80 households on average because some have 100, and others have 140. Let me tell you, when you see the money coming to your hands, you will be motivated to work harder, so they end up going the extra mile to make sure that they finish up all their households or at least up to 90% because of the fear of not being paid. I still owe it [poor performance] to the same reason - uncertainty of whether the money will come through, and I believe that those who are ill-motivated say, “aah, I might go and finish all my 140 households, and I do not get my incentive. *–* KII_Public Health Assistant

The demotivated CHWs ultimately carried out fewer home visits because they often lacked money for field coordination, including transport, airtime, and internet for those using mHealth devices.

Overall, the majority FGDs, one IDI, and most KIIs expressed a preference for cash over digital payments. This preference stemmed from the promptness and minimal delays associated with cash transactions, in contrast to digital payments, which were frequently delayed due to the verification process. Additionally, receiving cash payments necessitated all CHWs to gather centrally to sign for the cash individually, which mitigated payment inequities.

I will base on my interaction, not on my personal view but on my interaction [with CHWs]; they prefer cash. Because usually, they [CHWs] complain that the mobile money thing takes a little longer, like they have to verify the numbers, they have to verify the names. KII_IP_Public Health Asst_Male

I prefer cash because our bosses give us more reasons to delay. Moreover, the numbers registered in mobile money are not the same. Others are making mistakes. With cash, it is easy, so I prefer cash. If they can help us, they should continue with cash. IDI_CHW Leader_Male

Other participants mentioned that mobile money presented ‘ghost payment’ challenges, unlike cash payments:

The challenges we find in it is that you find the people who are sending money aren’t reliable [all laugh] there he shows that he has sent the money yet he has sent on his own phone on the other […] do you know that there are people whom we never trained within ICCM that have been getting salaries? FGD_CHWs_Mixed

## Discussion

This study analyzed the CHW performance across cash and digital payment modes independently and tested for statistical significance of differences. Furthermore, the factors associated with CHW performance were analyzed using the modified Poisson model for statistical significance to guide context-relevant recommendations. CHWs were found to have doubled their performance when they were paid cash. During the cash payment period, CHWs with higher education levels and those with side jobs performed significantly better than their counterparts. On the other hand, male CHWs, those with more years of experience, and CHWs with more than 60 households to supervise performed worse than their counterparts. Generally, cash payments were seen as more reliable than digital methods. Therefore, the choice of payment method should be tailored to the CHWs’ context, as it can significantly affect their morale to perform optimally.

The two-fold improved performance of CHWs, when they received cash, could be attributed to the prompt and reliable payments, which might have motivated them to effectively cover the duties assigned to them. When they received cash payments instead of digital payments, household visits conducted, and trainings attended by the CHWs doubled. The overall improved performance of CHWs, however, contradicted previous studies, which found better health worker performance with digital payments [34,36]. Given the poor livelihoods in refugee settlements, the meagre salary paid to the CHWs would perhaps ideally inspire their optimal performance if the salary was assured and prompt. However, the payment delays synonymous with digital methods likely demoralized the CHWs, reducing their zeal to perform optimally [25,48]. Given the contextual differences across these study settings, further research is needed to understand why this discrepancy exists. Nevertheless, health systems should recognize the importance of timely and assured payments in motivating frontline health workers. This may involve re-evaluating payment methods and addressing the contextual and structural issues related to payment delays in digital systems. This may involve regular assessment of payment systems and adjustments to address any identified shortcomings.

Delays in digital payment among health workers can result in demotivation, decreased job satisfaction, and potential disruptions in their ability to provide consistent and effective healthcare services [62,63]. Community health workers may be compelled to strike, abscond from duty or seek alternative employment opportunities with more reliable payment. The subsequent health workforce attrition and suboptimal performance affect the delivery and quality of routine services [44,64,65], such as household visits, vaccinations, and timely interventions. Diminished healthcare access can exacerbate the vulnerabilities of the refugee and host communities through increased morbidity and mortality [66,67]. Health systems may need to engage communities, develop outreach programs, and use targeted healthcare delivery models to improve health inequalities among vulnerable populations and access to timely and quality healthcare services. Health systems must adopt appropriate payment models to financially motivate CHWs to prevent attrition and poor health service delivery.

The CHWs with a higher education level performed better than their counterparts, as evidenced by previous studies in LMICs [18,19,43]. This finding could be attributed to highly educated CHWs having readily attained the necessary skills and bonding with the IPs. Their performance was much better than those with primary education or lower, and they attributed this success to receiving cash payments. CHWs believed that being highly educated added value to their work and made them more deserving of prompt compensation. With the digital payment delays and inequalities, highly educated CHWs felt undervalued, which led to sloppiness in attaining their targets. Health systems should address issues related to perceived value and recognition among CHWs, ensuring that education and skills are appropriately acknowledged. This may involve communication, recognition programs and fair compensation practices.

Similarly, during the cash payment period, CHWs with side jobs performed one and a half times better than those without side jobs. This was probably because those with side jobs were generally hard-working individuals who could dedicate more effort to their work since the payment was prompt. Secondly, they were less drawn towards their other side jobs since the payments from the CHW assignments were prompt and more assured. During the period of mobile payment, they were not motivated to work harder since payments were irregular and delayed. Thus, they diverted their attention to other jobs that pay cash. On the contrary, previous studies noted that CHWs with side jobs performed worse than those without [20,26]. Generally, the findings depicted that CHWs with side jobs were easily derailed from their duties, giving more attention to the better-rewarding side occupations. However, the studies did not disaggregate the findings across the modes of payment.

Future health systems should consider payment structures that align with CHW needs, preventing distractions from other occupations. More research is warranted on the context-appropriate modes of CHW incentivization in low-resource settings, including refugee settlements.

The study further revealed that during the mobile money payment period, the male CHWs performed worse than the females. This could be because women were more tolerant of the delays and irregularities linked to digital payments than men, who paid less attention to their duties when payments were delayed, subsequently failing to meet the set monthly targets. These results are similar to those from previous literature, which depicted female CHWs as better performers than males due to their perseverance and commitment [28]. Furthermore, men quickly neglected community health duties because of the uncertain rewards linked to digital payment methods, consequently opting for occupations with a more certain income to support their families [25,26]. This implies that health systems should acknowledge gender-related differences in response to CHW remuneration. Strategies may include tailored support mechanisms, addressing specific concerns of male CHWs and ensuring equitable compensation practices. On the other hand, recognizing and building on the strengths of female CHWs could contribute to a more resilient and dedicated community health workforce. Health systems should implement strategies that support and empower female CHWs, acknowledging their tenacity and vital contributions.

To address the challenges faced by male CHWs, health systems may need to explore ways to provide more financial certainty or incentives, ensuring that community health duties remain an attractive and viable option for both genders [68]. In addition, health systems should incorporate gender-sensitive strategies in the planning and management of community health programs through targeted training, support mechanisms and policy adjustments [28,69].

Additionally, CHWs who had served for more than 10 years performed worse than those with fewer years of experience, specifically during the digital payment period. On the other hand, previous studies associated more experienced CHWs with improved performance and productivity owing to the accumulated health knowledge, skill sets, and community trust [29,30], while others found no association at all [41]. This conflicting finding could be explained by the CHWs’ desolation and reduced morale arising from the irregularities of the digital payment method and the negative influence of poor remuneration on health workers’ performance [25,51].

Lastly, the association between poor CHW performance and an immense workload has been noted in previous studies [31–33]; in fact, this relationship is notably worse in emergency and humanitarian settings [7,21,22]. In this study, the association between performance and workload was more significantly observed when the CHWs were paid using mobile money. This implies that health systems need to consider the impact of workload on CHW performance, especially in emergency and humanitarian settings. Adequate support mechanisms, including timely and reliable payments, the provision of necessary resources, and the ability to address workload-related challenges, are essential for maintaining CHW motivation given the bulky assignments. The digital payment irregularities demotivated the CHWs, depriving them of the means to execute their duties effectively since they lacked airtime and transport to attend trainings. CHWs also lacked internet data bundles to submit surveillance reports promptly. The IPs did not facilitate such activities, yet they impact performance, as noted by a previous study [25].

### Study strengths and limitations

As far as we know, this study is the first to examine how payment methods affect the motivation of refugee CHWs. The main strength of this study lies in the fact that a mixed methods approach was used, ultimately providing more robust inference than qualitative or quantitative approaches. The quantitative data highlighted the counterfactual scenario when using either payment mode within the same settings, while the qualitative data delved into the contextual complexities influencing the findings. As a result, the study elucidated the appropriate intervention pathways for the developers of digital payment modalities within refugee settlements and similar settings.

While the multi-national setting of the refugee settlement presented language barriers, the study trained research assistants who were fluent in the local dialect (Swahili) and familiar with the local culture. Some responses were subject to social desirability bias, but the qualitative guide design maximally controlled for the bias through probing, data source triangulation, and assurance of anonymity. Some variables from the secondary database were excluded because of quality concerns, which could impact the overall generalizability of the study’s findings. However, the settlement stakeholders guided the selection of the variables relevant to the setting, increasing the internal validity and credibility of the study findings.

## Conclusions

The CHWs in this study performed better when paid by cash rather than with mobile money because cash payments were prompt and more reliable than digital payments. The timeliness and completeness of the payments significantly influenced on the motivation of CHWs to execute duties and subsequently affected their performance. Their motivation also varied according to personal characteristics such as education level, gender, experience, and assigned workload. Hence, attaining at least a secondary-level education was positively associated with improved performance during the cash payment period. Conversely, when digital payment was used, being male, serving longer than 9 years and being allocated to more than 60 households per month were negatively associated with the performance of CHWs.

## Recommendations

As health systems adapt to digital payments, it is important to gradually transition through a hybrid approach (cash and digital payment) while addressing the challenges associated with digital payment. In an effort to roll out digital payments globally, implementers should collaborate with service providers, such as electricity providers, telecom, and internet companies, to design digital payment methods that cater to financially constrained communities in hard-to-reach areas to overcome payment processing obstacles and delays highlighted by this study. Furthermore, consultative discussions between digital payment implementers and CHWs should be encouraged to promote the sharing of feedback, and clarification of payment-related concerns, hence fostering a sense of trust and providing an opportunity to identify solutions to payment challenges collaboratively. This can help reduce payment delays and enhance motivation among CHWs, which can improve service delivery among refugee populations.

## Data Availability

The dataset used for analysis can be availed upon reasonable request by writing an email to the corresponding author.
